# Evaluation of Antioxidant, Antidiabetic and Antiobesity Potential of Selected Traditional Medicinal Plants

**DOI:** 10.3389/fnut.2019.00053

**Published:** 2019-04-25

**Authors:** Satvir Sekhon-Loodu, H. P. Vasantha Rupasinghe

**Affiliations:** Department of Plant, Food, and Environmental Sciences, Faculty of Agriculture, Dalhousie University, Truro, NS, Canada

**Keywords:** phytochemicals, antioxidants, polyphenols, diabetes, amylase, glucosidase, obesity

## Abstract

This study evaluated potential antidiabetic and antiobesity properties *in vitro* of selected medicinal plants. The hot water (WE) and ethanol extracts (EE) of sweet gale (*Myrica gale* L.), roseroot (*Rhodiola rosea* L.), sheep sorrel (*Rumex acetosa* L.), stinging nettles (*Utrica dioica* L.) and dandelion (*Taraxacum officinale* L.) were tested for total antioxidant capacity using ferric reducing antioxidant power (FRAP) and DPPH^•^ scavenging capacity assays, followed by α-amylase, α-glucosidase and formation of advanced glycation end products (AGE) inhibition assays *in vitro*. Myrica gale EE had the highest total phenolic content (12.4 mmol GAE/L), FRAP value (17.4 mmol TE/L) and DPPH^•^ scavenging activity (IC_50_ = 3.28 mg/L). Similarly, Myrica gale also exhibited significantly lower IC_50_ values for the percentage inhibition of α-amylase (IC_50_ = 62.65 mg/L) and α-glucosidase (IC_50_ = 27.20 mg/L) compared to acarbose (IC_50_ = 91.71 mg/L; IC_50_ = 89.50 mg/L, respectively) (*p* ≤ 0.05). The 3T3-L1 preadipocyte study also revealed that Myrica gale EE (54.8%) and stinging nettles (62.2% EE; 63.2% WE) significantly inhibited the adipogenesis in adipocytes *in vitro* (*p* ≤ 0.05). Polyphenols present in these medicinal plants have the potential to use in managing type 2 diabetes and obesity.

## Introduction

Type 2 diabetes (T2D), characterized by hyperglycemia and abnormal carbohydrate metabolism, is a leading cause of morbidity and mortality worldwide and a major economic burden (1). According to the International Diabetes Federation, about 382 million people had diabetes in the year 2013, and the numbers are expected to get double by 2035 (2). These increased numbers of diabetes patients are mainly (>95%) T2D which is developed due to insulin resistance and pancreatic β-cell dysfunction, leading to hyperglycemia (3). The insulin resistance and pancreatic β-cell apoptosis can be traced back to obesity and also provide the link between T2D and obesity (4). The postprandial blood glucose levels have been found to play an important role in the onset and developing complications of T2D (5). One of the therapeutic strategies for managing postprandial hyperglycemia involves the inhibition of α-amylase and α-glucosidase (6). The synthetic drugs such as acarbose and miglitol have strong inhibitory action against α-amylase and α-glucosidase; however, they may result in abdominal distention, flatulence, vomiting, and diarrhea (7). The hyperglycemia can also result in the excessive non-enzymatic glycation of proteins and formation of advanced glycation end products (AGE). The glycation modifications can further deteriorate the pathology of diabetes by contributing to nephropathy, cataracts, vasculopathy, and atherosclerosis (8).

A number of studies and reviews have reported that phytochemicals such as phenolics offer potential therapeutic benefits in alleviating diabetes and obesity complications and inhibitory effects against α-amylase and α-glucosidase (9–13). A safer alternative for management of obesity and T2D are medicinal plants which help to maintain low blood glucose and prevent high blood pressure, as well as boost body antioxidant system and insulin regulation (14). Like many other metabolic disorders, T2D is also linked to obesity (15). Infusions and decoctions of traditional medicinal plants have been used for centuries to treat various health ailments including type 2 diabetes and obesity-related metabolic disorders (16). The water infusion prepared from wild raspberry (*Rubus idaeus* L.) stem exhibited the inhibition of α-amylase, α-glucosidase activities and anti-AGE activity (17). Dandelion (*Taraxacum officinale* L.) has been used in traditional cancer medicines due to its hydroxycinnamic acid derivatives and flavonoid glycosides content (18). The plants containing antioxidant compounds can protect β-cells from reactive oxygen species (ROS) and therefore, can prevent diabetes induced by ROS (14). Therefore, it is pertinent to explore the traditional medicinal plants, which can be utilized for lowering blood glucose levels and also obesity-related biomarkers.

This study aimed to assess the anti-diabetic activity *in vitro* of plant resources used in traditional medicine in north Newfoundland region of Canada. The extracts prepared from sweet gale (*Myrica gale* L*.)*, roseroot (*Rhodiola rosea* L.), sheep sorrel (*Rumex acetosa* L.), stinging nettles (*Utrica dioica* L.), and dandelion (*Taraxacum officinale* L.) were used to compare their antioxidant and antidiabetic properties *in vitro*. The study was extended further to explore and evaluate the antiadipogenic ability of selected plant extracts *in vitro* using 3T3-L1 preadipocytes.

## Materials and Methods

### Chemicals

The liquid chromatography standards: phloridzin, phloretin, chlorogenic acid, ferulic acid, and caffeic acid were purchased from Sigma Aldrich (Oakville, ON, Canada); catechin, epicatechin, quercetin, quercetin-3-*O*-galactoside and quercitin-3-*O*-glucoside from ChromaDex, Inc. (Santa Ana, CA, USA); quercitin-3-*O*-rhamnoside, quercitin-3-*O*-galactoside and anthocyanin standards from Indofine Chemical Company (Hillsborough, NJ, USA). The analytic solvents, chemicals, and enzymes were obtained from Sigma Aldrich (Oakville, ON, Canada). Mouse embryo 3T3-L1 cell line (ATCC, CL-173) was obtained from Cedarlane (Burlington, ON, Canada). Phenazine methosulfate (PMS) and 3-(4,5-dimethylthiazol-2-yl)-5-(3-carboxymethoxyphenyl)-2-(4-sulfophenyl)-2H-tetrazolium (MTS) were purchased from Sigma–Aldrich Canada Ltd. (Oakville, ON, Canada). Dulbecco's modified Eagle's medium (DMEM), penicillin-streptomycin solution, fetal bovine serum, bovine calf serum, trypsin-EDTA solution, dexamethasone (DEX), 3-isobutyl-1-methylxanthine (IBMX), insulin solution and all other cell biology grade chemicals were purchased from Sigma–Aldrich (Oakville, ON, Canada). Lipolysis colorimetric assay kit was purchased from Biovison Inc. (Milpitas, CA, USA) and adipogenesis assay kit from Cayman Chemical Co. (Ann Arbor, MI, USA).

### Plant Materials

All the plant materials used in this study were collected from the wild habitats of northern Newfoundland, Canada in the year 2015. After harvesting a bulk sample of approximately one kg from multiple growing locations, the samples were sub-divided into three replicates. The stems with leaves were cut into small chunks (1 cm) and were flash frozen in liquid nitrogen and freeze-dried in a freeze dryer (Kinetics, FTS Systems Inc, Stone Ridge, NY, USA) until constant weight was observed. The dried plant samples were ground in a coffee grinder (Model 6378-33, Sunbeam, China).

### Preparation of Hot Water Extractions (WE)

Five grams of freeze-dried samples were extracted in 100 ml of boiling water for 30 min (in triplicates), filtered through Whatman No. 4 paper under vacuum. The samples were reconstituted in dimethyl sulfoxide (DMSO). The supernatants were stored at −80°C until further use for various assays.

### Preparation of Ethanolic Extractions (EE)

Five grams of each sample was sonicated in 100 mL of absolute ethanol, three times for 15 min with 10 min intervals in between sonications (in triplicates). After the extraction, extracts were filtered separately using Whatman No. 1 filter papers under vacuum. The extracts were concentrated using a rotary evaporator (Rotavapor, R-200, Buchi, Flawil, Switzerland) at 37°C, followed by nitrogen drying to evaporate ethanol. The extracts were then stored at −80°C until further use.

### UPLC-MS/MS Analysis of Phenolic Compounds

Analyses of major individual phenolic compounds present in freeze-dried plant extracts were performed according to a previously reported method (19). All analyses were conducted using ultra-pressure liquid chromatography (Waters, Milford, MA, USA) coupled with Micromass Quattro micro API tandem mass spectrometry (UPLC-MS/MS) system and controlled with Mass Lynx V4.0 data analysis system (Micromass, Cary, NC, USA). The column used was Aquity BEH C_18_ (100 × 2.1 mm, 1.7 μm) (Waters, Milford, MA, USA). For the separation of the flavonol, flavan-3-ol, phenolic acid and dihydrochalcone, the mobile phase consisted of 0.1% formic acid in water (solvent A) and 0.1% formic acid in acetonitrile (solvent B). A linear gradient profile was used with the following proportions of Solvent A applied at time t (min); (t, A%): (0, 94%), (2, 83.5%), (2.61, 83%), (2.17, 82.5%), (3.63, 82.5%), (4.08, 81.5%), (4.76, 80%), (6.75, 20%), (8.75, 94%), and (12, 94%).

Electrospray ionization in negative ion mode (ESI-) was used for the analysis of the flavonol, flavan-3-ol, phenolic acid, and dihydrochalcone; the following conditions were used: capillary voltage 3,000 V, nebulizer gas (N_2_) temperature 375°C at a flow rate of 0.35 mL/min. The settings for positive ion experiments were as follows: capillary voltage (25–50V) was optimized for each individual compound. Multiple reaction-monitoring (MRM) mode using specific precursor/product ion transitions was employed for quantification in comparison with standards: m/z 301→ 105 for quercetin (Q), m/z 609→ 301 for Q-3-*O*-rutinoside, m/z 463→ 301 for Q-3-*O*-glucoside and Q-3-*O*-galactoside, m/z 448→ 301 for Q-3-*O*-rhamnoside, m/z 595→ 301 for Q-3-*O*-peltoside, m/z 273→ 167 for phloritin, m/z 435→ 273 for phloridzin, m/z 353→ 191 for chlorogenic acid, m/z 179→ 135 for caffeic acid, m/z 193→ 134 for ferulic acid and isoferulic acid, m/z 289→ 109 for catechin, m/z 290→ 109 for epicatechin, and m/z 305→ 125 for epigallocatechin. In MRM experiments, both quadrupoles were operated at unit resolution.

### Total Phenolic Content (TPC) by Folin-Ciocalteu Reagent Assay

TPC was measured by Folin-Ciocalteu (FC) reagent using the method described by Singleton and Orthofer (20). Folin-Ciocalteu reagent (0.2 N) and 7.5% (w/v) sodium carbonate (Na_2_CO_3_) was prepared. At the first step, 20 μL of samples were added into 96-well microplate, and 100 μL of FC reagent was added to each well. After 5 min, 80 μL of Na_2_CO_3_ were added, and the microplate was placed in the dark at room temperature for 2 h. The absorbance of resultant color was read at 760 nm using a FLUOstar OPTIMA plate reader (BMG Labtech., Durham, NC, USA) and TPC values were expressed as mg gallic acid equivalents (GAE) per liter.

### Ferric Reducing Antioxidant Power (FRAP) Assay

The FRAP was conducted based on the method described by Benzie and Strain (21) with some modifications (19). The reaction reagent was made freshly by mixing 300 mM acetate buffer (pH 3.6), 10 mM TPTZ solution, and 20 mM FeCl_3_ solution in the ratio of 10:1:1. The TPTZ solution was prepared on the same day of analysis. The Trolox standard solution (1 mM) was prepared in ethanol. Samples (20 μL) were taken in the 96-well microplate (COSTAR, 9017). The microplate with samples was incubated at 37°C for 15 min. The working reagent (180 μL) was injected into the sample wells by the plate reader programmed pump. The absorbance was read at 595 nm using FLUOstar OPTIMA plate reader. FRAP values were expressed as mg Trolox equivalents (TE) per liter.

### DPPH^·^ Radical Scavenging Activity

The assay was performed using the method of Shimada et al. (22) with some modifications (19). DPPH solution was prepared in 95% ethanol, 100 μL of this solution were mixed with 100 μL of sample in 96-well microplate. Various concentrations (20, 40, 60, 80, and 100 μL) of the extracts were used. The mixture was incubated for 30 min at room temperature. The absorbance of resulted color was read at 517 nm using the plate reader. The DPPH^**·**^ scavenging activity was expressed as an IC_50_ value (the concentration of the antioxidant required to scavenge 50% of DPPH^**·**^ present in the test solution.

### α-Amylase Inhibitory Assay

The pancreatic porcine α-amylase assay was adapted from Sudha et al. (23) with modifications (17). Briefly, both EE and WE were freeze-dried to obtain a completely dry powder. The test extracts enzyme and soluble starch were dissolved in 20 mM sodium phosphate buffer containing 6 mM NaCl (pH 6.9). The test extracts at different concentrations were dissolved in the buffer solution. To a test tube, 250 μL of pancreatic porcine α-amylase (1 U/mL, dissolved in the buffer (pH 6.9) and 100 μL of test extract at a concentration ranging from 15.6 to 250 mg/L were added. The mixture was pre-incubated at 37°C for 15 min, before the addition of 250 μL of 0.5% starch. The mixture was then vortexed and incubated again at 37°C for 15 min followed by the reaction termination using 1 mL of dinitrosalicylic acid color reagent. The tubes were placed in a boiling water bath for 5 min, cooled to room temperature and diluted. Two hundred microliters of the reaction mixture were taken into a 96-well clear plate, and the absorbance was read at 540 nm using the FLUOstar OPTIMA plate reader. The control α-amylase at 1 U/mL without any inhibitor represented 100% enzyme activity. Appropriate test extract controls containing the reaction mixture except the enzyme were used to correct for the color interference. A known α-amylase inhibitor, acarbose was used for comparison studies. The percentage inhibition of the test sample on α-amylase was calculated as:

$$\begin{array}{l} Inhibition\;\left(\%\right)=100\times \left(AC-AS\right)/\left(AC\right) \end{array}$$

Where AS, AC is the absorbance of sample and control, respectively. The results were expressed in terms of IC_50_ representing the concentration of test extracts required to cause the enzyme inhibition by 50%.

### α-Glucosidase Inhibitory Assay

The α-glucosidase inhibitory assay was adapted from Li et al. (24) with modification. Briefly, both EE and WE were freeze-dried to obtain a completely dry powder. From this powder, various concentrations of each extract were prepared in 10 mM potassium phosphate buffer (pH 6.8). To a 96-well clear plate, a reaction mixture containing 20 μL extract at different concentrations, 20 μL α-glucosidase (0.5 U/mL) and 60 μL of 10 mM potassium phosphate buffer (pH 6.8) were pre-incubated at 37°C for 15 min before adding 20 μL of 5 mM p-nitrophenol-α-D-glucopyranoside substrate. The mixture was then incubated at 37°C for the reaction to take place. After 15 min, 80 μL of stop solution containing 200 mM sodium carbonate was added. Then the absorbance at 405 nm was recorded using the microplate reader. The positive control sample was the mixture of the enzyme and substrate without inhibitors. The sample controls and blanks were the mixtures of sample and control, respectively, except α-glucosidase was instead with buffer, respectively. The inhibition (%) of the test sample on α-glucosidase was calculated same way as with α-amylase assay. Acarbose, a prescribed drug for α-glucosidase inhibition, was also used for comparison purpose.

### Inhibition of AGE Formation

The procedure was based on previous methods (25) with minor modifications (17). The incubation mixtures are in a final volume of 1.0 ml, containing Bovine serum albumin (BSA, 5.0 mg/ml) and D-glucose (36 mg/ml) and vehicle (Control) or the inhibitor at 0.01, 0.02, 0.04, 0.05, 0.1, 0.2, 0.5, 1.0 mg/ml concentrations or aminoguanidine (AG, 1.0 mg/ml). Aminoguanidine, a known AGE formation inhibitor, was used as a positive control. All these solutions were dissolved in 0.2 M phosphate buffer saline (pH 7.4) containing sodium azide (0.02% w/v). The mixtures in screw-capped test tubes were incubated in triplicate in an incubator at 37°C for a week. Fluorescent AGE was monitored on a microplate reader using 340 and 420 nm as the excitation and emission wavelengths. Experiments were conducted in triplicates. Percentage of the AGE inhibition was calculated by the following equation:

$$\begin{array}{l} inhibition\left(\%\right)=\left[1-\left(\frac{Fluorescence\;of\;the\;test}{Fluorescence\;of\;control}\right)\right]\times 100 \end{array}$$

### 3T3-L1 Cell Culture

Mouse embryo 3T3-L1 cell line (ATCC, CL-173) was obtained from Cedarlane (Burlington, ON, Canada). The 3T3-L1 preadipocytes were grown in Dulbecco's Modified Eagle's Medium (DMEM) supplemented with 10% fetal bovine serum (FBS). The culture was maintained at 37°C in a humidified atmosphere, containing 5% CO_2_. For adipocyte differentiation, the 3T3-L1 cells were grown in 96-well plates (0.2% gelatin-coated) at cell density 2.0 × 10^4^ cell/well to confluence for 48 h. After incubating, preadipocytes were stimulated by a differentiation medium containing l μM dexamethasone (DEX) (Sigma Aldrich, Canada), 0.5 mM 3-isobutyl-1-methylxanthanine (IBMX) (Sigma Aldrich, Canada) and 1 μg/ mL insulin in DMEM with 10% FBS. After 2 days the medium was replaced with DMEM containing 10% FBS and 1 μg/mL insulin and corresponding treatment compounds were added for further analysis.

### Adipocyte Cell Viability Assay

Cell viability was determined using the 3-(4,5-dimethylthiazol-2-yl)-5-(3-carboxymethoxyphenyl)-2-(4-sulfophenyl)-2H-tetrazolium (MTS) assay (26). The 3T3-L1 preadipocytes were grown in 96-well plates at the cell density of 2.5 × 10^4^/well and treated with 50 and 100 μg/mL of plant extracts concentrations for 48 h at 37°C. The cells were incubated in the presence of MTS and PMA for 3 h at 37°C. The absorbance was measured at 570 nm using a FLUOstar OPTIMA plate reader (BMG Labtech, Durham, NC, USA).

### Determination of Lipid Accumulation by Oil Red O Staining

The Oil red O assay was conducted on differentiated adipocytes on day 10 in 96-well plate using adipogenesis assay kit from Cayman Chemicals (Ann Arbor, MI, US). The medium was removed from the wells and cells were fixed with 75 μL lipid droplet assay fixative. After 15 min incubation, the wells were washed five times with 100 μL wash solution. The Oil red O working solution (75 μL) was added to the wells and incubated for 20 min. The Oil red O solution was removed at the end of incubation, and the wells were washed with wash solution until no visible pink color in the wash. The pictures were taken under a microscope. The dye was extracted with 100 μL dye extraction solution. The absorbance was read at 490 nm using a FLUOstar OPTIMA plate reader (BMG Labtech, Durham, NC, USA).

### Statistical Analysis

All the experiments were performed in triplicates, and complete randomized designs were used. The normality of the residuals was tested using the Anderson-Darling test. The variables were tested using one-way analysis of variance (ANOVA), and the multiple mean comparisons were performed in SAS V8, Cary, NS, USA) with Tukey's test. The data are expressed as a mean ± standard deviation.

## Results and Discussion

### Total Phenolic Content and Antioxidant Capacity of Hot Water and Ethanol Extracts

**Figure 1:**
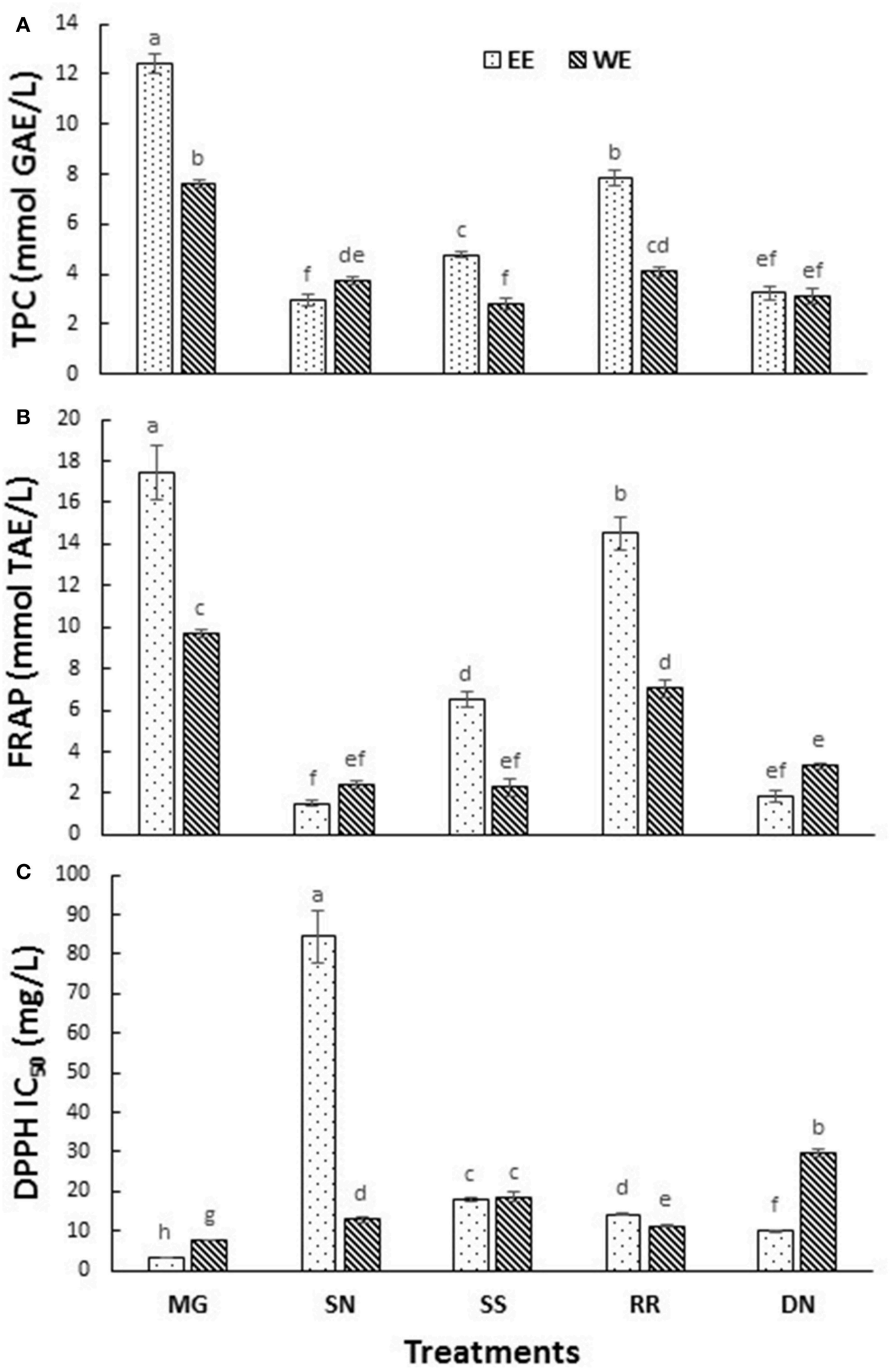
The total phenolic content **(A)**, FRAP **(B)**, and DPPH radical scavenging activity **(C)** measured in the tested hot water and ethanol plant extracts. Values are the mean ± SD of triplicate experiments. Different letters denote significant differences (*p* ≤ 0.05, Tukey's test). MG, Myrica gale; SN, stinging nettles; SS, sheep sorrel; RR, roseroot; DN, dandelions.

The postprandial blood glucose level is the primary factor to maintain in the management of T2D. The current antihyperglycemic strategies include 11 major classes of antidiabetic agents [sulfonylureas, meglitinides, biguanides, thiazolidinediones, α-glucosidase inhibitors, glucagon-like peptide-1 receptor agonists, dipeptidyl peptidase-IV inhibitors, amylin analogs, dopamine D2-receptor agonists, bile acid sequestrants and insulin (3)]. The drug intervention has also been associated with inexorable side effects, mainly hypoglycemia, weight gain and gastrointestinal discomfort (7). The antioxidant effects attributed to the polyphenols of plants plays a pivotal role in protecting the body from oxidative stress, diabetes, cardiovascular diseases, and cancer. Therefore, the plant-based therapeutics is a potential alternative therapy to explore due to their reported safety and nutraceutical benefits (27). To compare the total phenolics and antioxidant capacity of the EE and WE of five medicinal plant material, TPC (Folin Caiocalteu), FRAP, and DPPH radical scavenging assays were performed (Figure 1). The total phenolic content was the highest in Myrica gale EE (12.4 mmol GAE/L), followed by roseroot EE (7.8 mmol GAE/L). Roseroot plant is rich in flavonoids and phenolic glycosides: salidrosides and rosavins (28). Similarly, Myrica gale (17.4 mmol TE/L) and roseroot (14.5 mmol TE/L) had greater FRAP values in corresponding EE. Total phenolic content and FRAP values were noticeably high in EE compared to WE. The DPPH radical scavenging activity was highest in EE of Myrica gale, roseroot, dandelion and roseroot WE (IC_50_ = 3.3, 14.2, 9.8, 11.3 mg/L, respectively). Similar to our finding, Myrica gale ethanol extracts have been reported to exert strong antioxidant capacity due to the presence of phenolic compounds (Table 1) (29). The reports are scarce on the identification of phenolic compounds in Myrica gale stem and leaves. Our findings indicate that the overall antioxidant capacity was higher in EE as compared to the corresponding WE.

**Table 1:** UPLC-MS/MS quantification of selected major phenolic compounds (mg/L) present in ethanol and hot water plant extracts.

**Phenolic Group**	**MG**	**SN**	**SS**	**RR**	**DN**
**ETHANOL EXTRACT**					
Flavonols	223.2 ± 1.2	7.6 ± 0.1	148.6 ± 4.4	32.0 ± 0.6	5.3 ± 0.1
Dihydrochalcones	2.5 ± 0.0	ND	1.8 ± 0.1	0.1 ± 0.0	0.1 ± 0.0
Phenolic acids	12.0 ± 0.4	20.1 ± 0.2	145.2 ± 18.2	4.1 ± 0.2	85.6 ± 0.9
Total Phenolics (UPLC-MS/MS)	237.7	27.7	295.6	36.2	91
**HOT WATER EXTRACT**					
Flavonols	133.4 ± 1.9	1.5 ± 0.1	51.0 ± 3.4	14.9 ± 0.2	0.1 ± 0.3
Dihydrochalcones	ND	ND	ND	0.1 ± 0.1	ND
Phenolic acids	7.7 ± 0.6	37.1 ± 0.5	82.3 ±6.9	3.8 ± 0.1	0.4 ± 0.2
Total Phenolics (UPLC-MS/MS)	141.1	38.6	133.3	19.6	0.6

*The phenolic groups include flavonols (Quercetin-3-O-galactoside, Quercetin-3-O-glucoside, Quercetin-3-O-rhamnoside, Quercetin-3-O-rutinoside, Quercetin); Dihydrochalcones (phloridzin, phloritin); Phenolic acids (Chlorogenic acid, Caffeic acid, Ferulic acid). MG, Myrica gale; SN, stinging nettles; SS, sheep sorrel; RR, roseroot; DN, dandelions; ND, not detected*.

### Antidiabetic Activity *in vitro*

**Figure 2:**
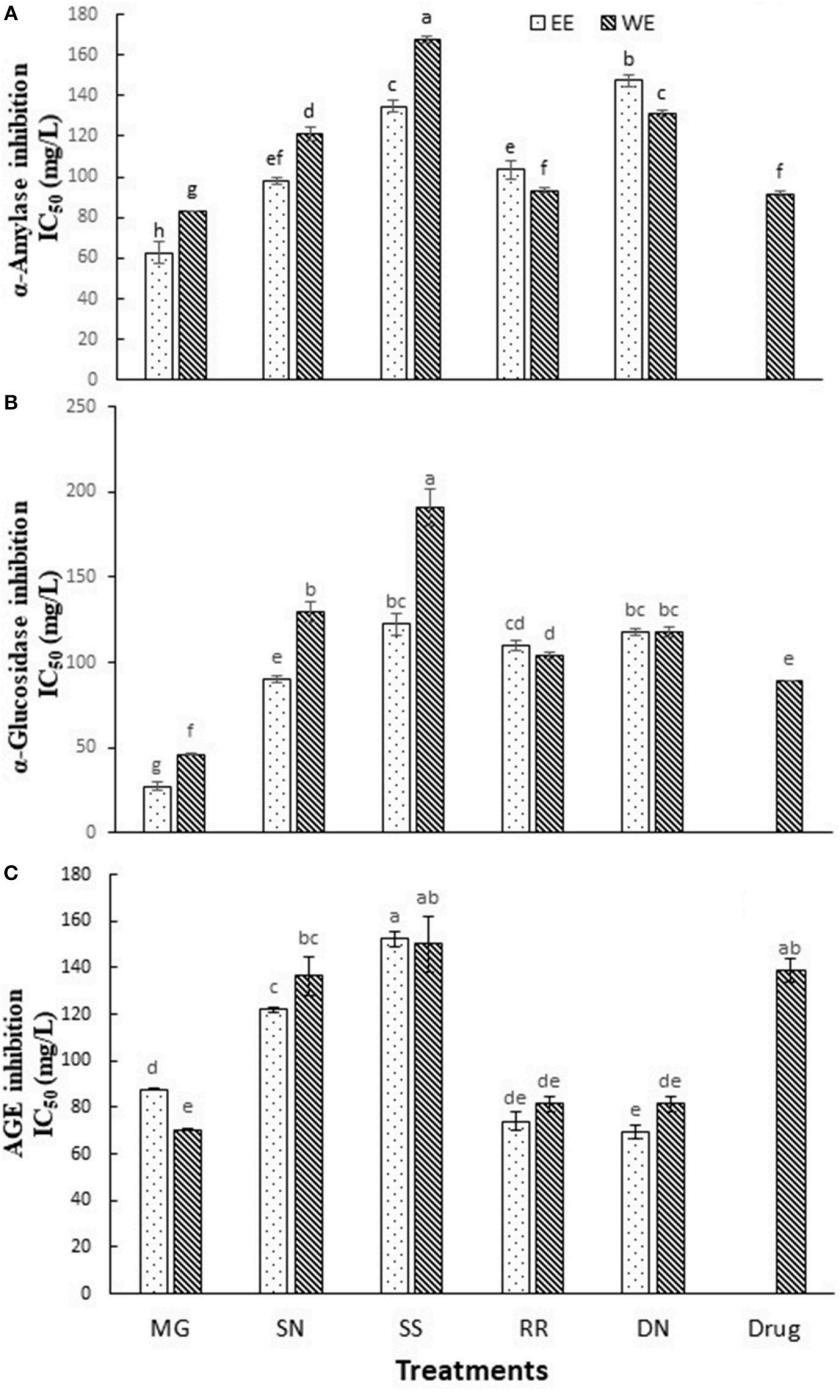
The inhibition of α-amylase **(A)**, α-glucosidase **(B)**, and AGE **(C)** by the tested hot water and ethanol plant extracts. Values (IC_50_) are the mean ± SD of triplicate experiments. Different letters denote significant differences (*p* ≤ 0.05, Tukey's test). MG, Myrica gale; SN, stinging nettles; SS, sheep sorrel; RR, roseroot; DN, dandelions.

Dietary polyphenols, in addition to their antioxidant effects, have been reported to exert anti-hyperglycemic effects by binding to glucose transporters (3) and competitive inhibition of digestive enzymes (16). Carbohydrate hydrolyzing enzymes, α-amylase and α-glucosidase digest dietary starch and degrade the oligosaccharides to glucose, resulting postprandial glucose surge. Therefore, inhibition of α-amylase and α-glucosidase activities is one of the primary approaches to manage hyperglycemic conditions of T2D patients. Acarbose is the most commonly prescribed α-glucosidase inhibitory drug. The inhibition of α-amylase, α-glucosidase activity and AGE (IC_50_ values) by the tested plant extracts is described in Figure 2. Myrica gale ethanol extract exhibited the highest inhibition of α-glucosidase with IC_50_ values (27 mg/L) which was significantly efficacious compared to the drug, acarbose IC_50_ value (α-glucosidase, 89 mg/L). The polyphenols are known to interact with the enzyme through non-specific binding, leading to inhibition of enzyme activity. The polyphenols tend to more effective on α-glucosidase inhibition with increase with molecular weight and degree of polymerization (30).

The extracts of dandelion (EE), roseroot (EE) and Myrica gale (WE) exhibited significant inhibition of formation of AGE (IC_50_ = 69.4, 74.0, 70.4 mg/L, respectively) (Figure 2C), compared to common anti-glycation drug aminoguanidine (IC_50_ = 138 mg/L). A previous study has reported that wild raspberry stem and blackberry stem extracts had stronger anti-glycation properties than aminoguanidine (17). Dandelion is used in traditional anticancer and antimicrobial medicines; however, only a few reports are available on its phytochemical analysis. According to the previous studies, dandelion shoots contain hydroxycinnamic acid, luteolin and flavonoid glycosides (18, 31). Dandelion phenolic fraction showed an inhibitory action on H_2_O_2_ and H_2_O_2_/Fe-induced oxidation in human plasma *in vitro* (18) and protected against oxidative stress linked atherosclerosis *in vivo* (32).

### Correlation Analysis of Total Phenolics, Antioxidant Capacity, and Antidiabetic Activity

Plant polyphenols could modulate various enzymes and immune cells in the human body, apart from their antioxidant potential (33, 34). Pearson correlation was applied to total FC phenolics, DPPH^•^ scavenging activity, FRAP, α-amylase and α-glucosidase activity (Table 2). DPPH^•^ scavenging activity exhibited significant non-linear correlation with TPC (*r* = −0.55; *p* = 0.33, *r* = −0.68; *p* = 0.25), FRAP (*r* = −0.57; *p* = 0.30, *r* = −0.63; *p* = 0.27), a linear correlation with α-amylase in WE (*r* = 0.60; *p* = 0.27), α-glucosidase activity (*r* = 0.09; *p* = 0.88; *r* = 0.46; *p* = 0.44) and AGE (*r* = 0.41; *p* = 0.48; *r* = 0.07; *p* = 0.90) in EE and WE, respectively. Similarly, a negative correlation of DPPH^•^ scavenging activity was reported with FRAP and TPC (17). TPC and AGE exhibited significant negative relationship in both EE and WE (*r* = −0.31; *p* = 0.60; *r* = −0.59; *p* = 0.29). However, UPLC-MS/MS total phenolics depicted a significant linear correlation with AGE in EE (*r* = 0.50; *p* = 0.38) and non-linear relationship in WE (*r* = −0.46; *p* = 0.43). There was no significant correlation between α-glucosidase and α-amylase activity (*p* < 0.05) in both EE and WE; however, FRAP values demonstrated significant negative correlation with α-amylase (*r* = −0.69; *p* = 0.27, *r* = −0.86; *p* = 0.06), α-glucosidase activity (*r* = −0.61; *p* = 0.28; *r* = −0.86; *p* = 0.06) and AGE (*r* = −0.31; *p* = 0.61; *r* = −0.78; *p* = 0.12) in EE and WE, respectively. The AGE values in both EE and WE showed a significant linear correlation with α-amylase and α-glucosidase (*r* = 0.14; *p* = 0.82; *r* = 0.79; *p* = 0.11, *r* = 0.19; *p* = 0.75; *r* = 0.85; *p* = 0.07, respectively). However, there was no significant correlation between TPC, FRAP, α-glucosidase activity (*p* < 0.05) in EE. On the other hand, TPC demonstrated a strong negative correlation with α-amylase (*r* = −0.78; *p* = 0.12) and α-glucosidase activity (*r* = −0.87; *p* = 0.06) in hot WE. The reducing power of phenolics based on their ability to reduce Fe^3+^ to Fe^2+^ is a potent antioxidant mechanism and express the concentration of electron donating compounds. The UPLC-MS/MS total phenolic content showed significant linear correlation (*r* = 0.29; *p* = 0.63; *r* = 0.79; *p* = 0.11) with FRAP values. TPC showed significant linear correlation in EE (*r* = 0.37; *p* = 0.53), however no significant correlation was observed in WE. This might be attributed to the phenolic compounds under detection in UPLC-MS/MS analysis. The free radical quenching ability is an important link to prevent the oxidative stress related to hyperglycemia (35). The enzyme inhibitory activity was not linearly correlated to TPC of the extracts; however, it was associated with individual type of phenolic compound (9). ROS are generated during process of protein glycation and diabetic patients show elevated levels of iron and copper ions. The phenolic compounds have been reported to scavenge radicals generated in the glycation process and therefore, DPPH^•^ scavenging activity and inhibition of AGE showed a strong correlation for polyphenols-rich plant extracts (36). The present correlation analysis indicates that α-amylase, α-glucosidase, and AGE inhibition ability of the studied EE and WE could be attributed to their FRAP and DPPH^•^ scavenging properties.

**Table 2:** Pearson correlation coefficients to exhibit linear relationship among the total phenolic compounds, FRAP, DPPH, α-amylase, α-glucosidase, and AGE in ethanol and hot water plant extracts.

**Extract**	**Total FC phenolics**	**FRAP**	**DPPH**	**α-Amylase**	**α-Glucosidase**	**AGE**
**ETHANOL**						
FRAP	0.95					
DPPH	−0.55^*^	−0.57^*^				
α-Amylase	−0.78^*^	−0.69^*^	−0.08^*^			
α-Glucosidase	−0.78	−0.61^*^	0.09^*^	0.90		
AGE	−0.31^*^	−0.31^*^	0.41^*^	0.14^*^	0.19^*^	
UPUPLC-MS/MS /MS total phenolics	0.37^*^	0.29^*^	−0.49^*^	−0.04^*^	−0.24^*^	0.50^*^
**HOT WATER**						
FRAP	0.89					
DPPH	−0.68^*^	−0.63^*^				
α-Amylase	−0.78^*^	−0.86^*^	0.60^*^			
α-Glucosidase	−0.87^*^	−0.86^*^	0.46^*^	0.93		
AGE	−0.59^*^	−0.78^*^	0.074^*^	0.79^*^	0.85^*^	
UPUPLC-MS/MS/MS total phenolics	0.98	0.79^*^	−0.69^*^	−0.70^*^	−0.81^*^	−0.46^*^

* *Significant correlations (p ≤ 0.05). FC, Folin-Ciocalteu; FRAP, ferric reducing antioxidant power; AGE, advanced glycation products; DPPH, 1, 1-diphenyl-2-picrylhydrazyl radical scavenging activity (IC_50_); UPLC-MS/MS, ultra-pressure liquid chromatography-mass spectrometry*.

### Antiadipogenic Effect of Test Compounds in 3T3-L1 Preadipocytes

**Figure 3:**
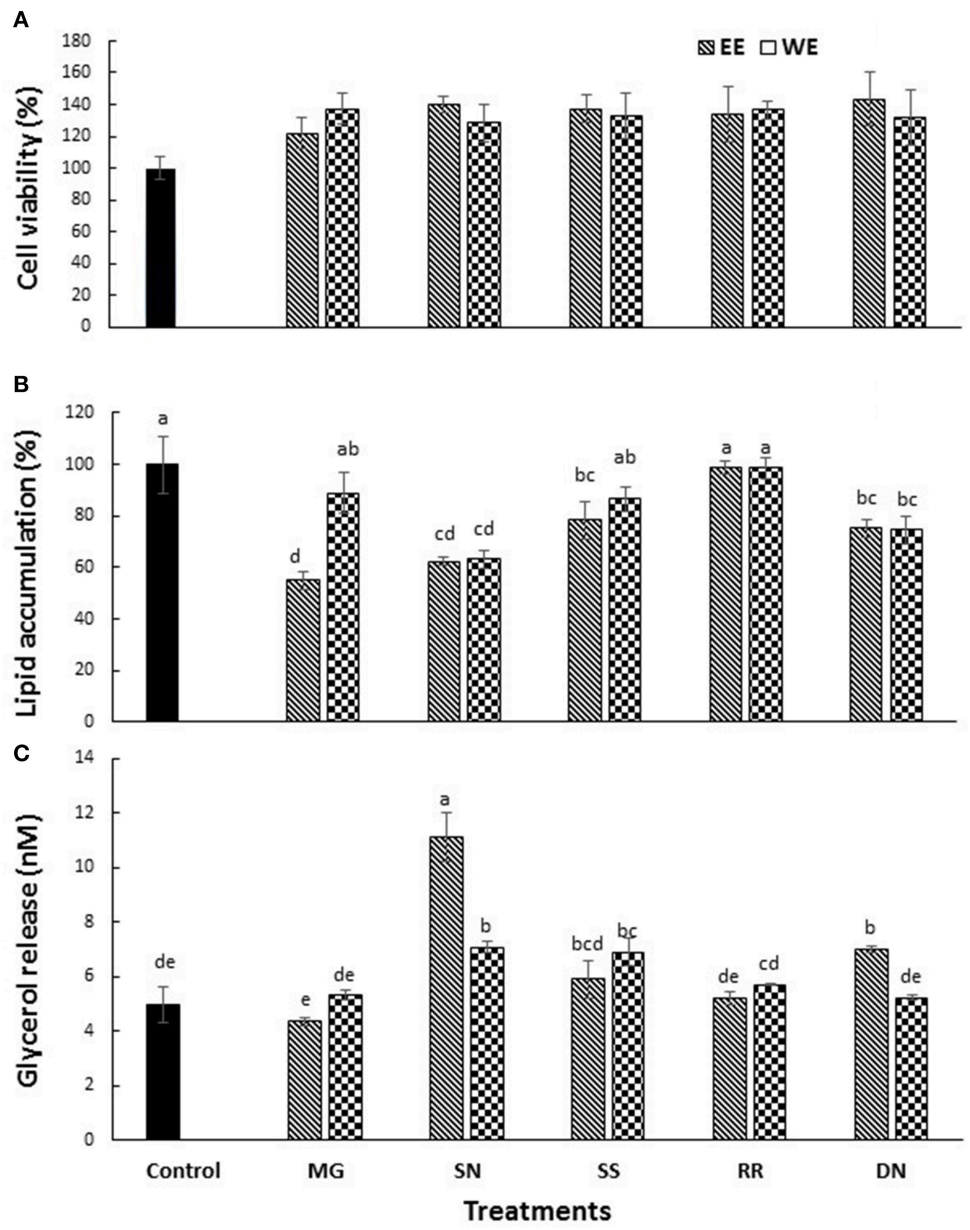
The effect of plant extracts on cell viability **(A)**, adipogenesis measured by % lipid accumulation **(B)**, and glycerol release **(C)** in 3T3-L1 adipocytes. The 3T3-L1 cells were treated with five test plant extracts (50 μg/mL) for 48 h to measure the cell viability. Differentiation was induced in 3T3-L1 pre-adipocytes two-days post-confluence. The cells were treated with plant extracts (50 μg/ml) for 7 days for lipolysis assay, and glycerol release was measured. The lipid content was analyzed on day 10 post-confluence. Values are the mean ± SD of triplicate experiments. Different letters denote significant differences (*p* ≤ 0.05, Tukey's test). MG, Myrica gale; SN, stinging nettles; SS, sheep sorrel; RR, roseroot; DN, dandelions.

The cytotoxicity of EE and WE of Myrica gale, stinging nettles, sheep sorrel, roseroot and dandelion (50, 100 μg/ mL) was determined using MTS assay. Cell viability was above 80% for the concentration (50 μg/ mL) of treatment compounds used in this study (Figure 3A).

**Figure 4:**
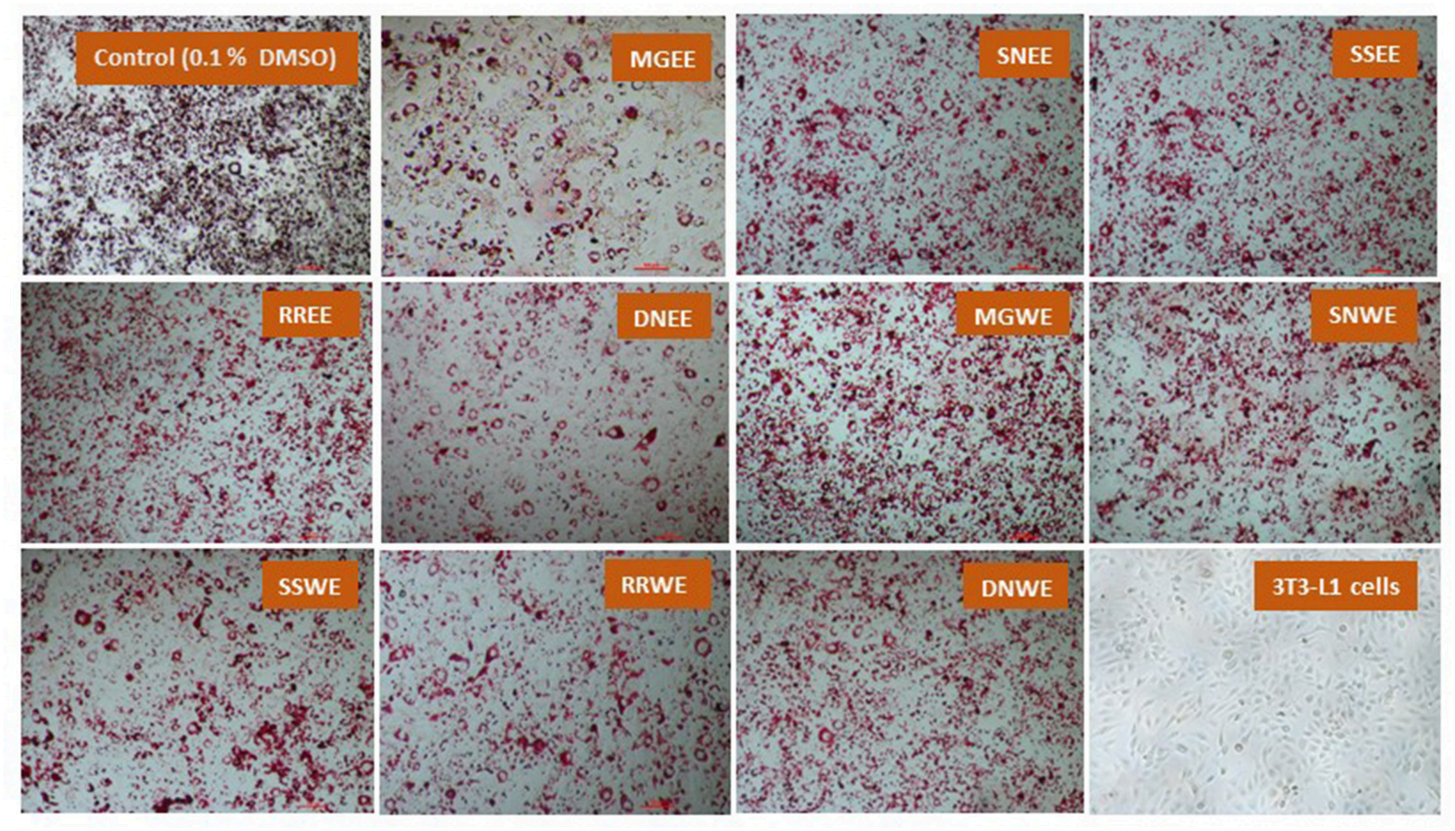
The effect of plant extracts on adipogenesis in differentiated 3T3-L1 preadipocytes (day 10) using Oil red O assay. The concentration of the extracts was 50 μg/ mL. MG, Myrica gale; SN, stinging nettles; SS, sheep sorrel; RR, roseroot; DN, dandelions; EE, ethanolic extract; WE, hot water extracts.

Obesity is characterized by an increase in fat cells along with an increase in the size of lipid droplets in the cells, as a result of adipogenesis. Therefore, one of the target anti-obesity mechanism is to increase lipolysis and decrease adipogenesis (15). The influence of native medicinal plant extracts on the regulation of adipogenesis and lipolysis in 3T3-L1 cells was analyzed. The microscopic images of cells stained with Oil red O dye showed that size of lipid droplets was smaller in Myrica gale (EE), stinging nettles (EE, WE), sheep sorrel (EE), and dandelion extracts (EE, WE) in Figure 4. The intracellular lipid accumulation was measured by Oil red O assay to compare the % adipogenesis between treatments in Figure 3B. Myrica gale EE treatment had significantly lower adipogenesis (54.8%; *p* ≤ 0.05), followed by stinging nettles extracts (62.2–63%; *p* ≤ 0.05) and dandelions extracts (74.5–75.2%; *p* ≤ 0.05) compared to the control (0.1% DMSO). However, stinging nettles EE demonstrated significantly higher lipolysis (11.1 nmol glycerol release) compared to the control (4.9 nmol), followed by stinging nettles WE (7.0 nmol), sheep sorrel EE and WE (5.9, 6.9 nmol), and dandelion EE (6.9 nmol) (*p* ≤ 0.05) (Figure 3C). The stinging nettles EE and WE have mainly phenolic acids according to the UPLC-MS/MS analysis and WE also exhibited high DPPH^•^ scavenging ability. Therefore, the results suggest that phenolic acids might have the ability to induce lipolysis by scavenging ROS. Water-soluble phenolics have been reported to have similar ROS scavenging ability and lipolysis potential (37). Several *in vivo* studies have revealed that increasing lipolysis in adipocytes does not increase circulating fatty acids and facilitate fatty acid oxidation within the adipocytes (38, 39). Similar to our results, another research study on *Momordica charantia* plant extracts has reported that inhibition of adipogenesis might not be related to increasing in lipolysis (38). Hence, the decrease in glycerol release may be linked to decreased intracellular lipids in Myrica gale treatments infusion.

## Conclusion

The present study provides the first pharmacological insight into the antioxidant and antidiabetic potential of the selected Canadian traditional medicinal plants. Myrica gale and roseroot extracts showed high antioxidant capacity. These traditional medicinal plant extracts also reduced significantly α-amylase and α-glucosidase activities compared to the most common drug, acarbose, indicating that the polyphenols present in the extracts have potential to reduce postprandial hyperglycemia by delaying the carbohydrate digestion. The antidiabetic ability to inhibit α-amylase, α-glucosidase enzymes, and AGE are needed to be further explored using *in vivo* experimental models to validate the findings in the present study. The present finding would be useful for future research directions on the application of traditional medicinal plants in the development of nutraceuticals and pharmaceuticals.

## Author Contributions

SS-L conducted all the experiments, data analyzing and writing the first draft of the manuscript. HR, the principal investigator, contributed by advising methods and experimental design, supervising data analysis and reviewing the manuscript.

### Conflict of Interest Statement

The authors declare that the research was conducted in the absence of any commercial or financial relationships that could be construed as a potential conflict of interest.
